# Prevalence Rate and Molecular Characteristics of *Oestrus ovis* L. (Diptera, Oestridae) in Sheep and Goats from Riyadh, Saudi Arabia

**DOI:** 10.3390/ani11030689

**Published:** 2021-03-04

**Authors:** Dina M. Metwally, Shurug A. Albasyouni, Ibrahim A.H. Barakat, Isra M. Al-Turaiki, Amal M. Almuhanna, Muhammad A. Bashir, Hanadi B. Baghdadi, Manal F. El-Khadragy, Reem A. Alajmi

**Affiliations:** 1 Zoology Department, College of Science, King Saud University, P.O. Box 2455, Riyadh 11451, Saudi Arabia; shurugaalbasyouni@gmail.com (S.A.A.); ibrahimahb@yahoo.com (I.A.H.B.); aalmuhanna@KSU.EDU.SA (A.M.A.); ralajmi@ksu.edu.sa (R.A.A.); 2 Parasitology Department, College of Veterinary Medicine, Zagazig University, Zagazig 44519, Egypt; 3 Cell Biology Department, National Research Center, 33 Bohouth St., Dokki, Giza 12622, Egypt; 4 Department of Information Technology, College of Computer and Information Sciences, King Saud University, Riyadh 11451, Saudi Arabia; ialturaiki@ksu.edu.sa; 5 Department of Plant Protection, Faculty of Agricultural Science Ghazi University, Dera Ghazi Khan 32200, Pakistan; abashir@gudgk.edu.pk; 6 Biology Department, College of Science, Imam Abdulrahman Bin Faisal University, Dammam City 31441, Saudi Arabia; hbbaghdadi@iau.edu.sa; 7 Basic and Applied Scientific Research Center, Imam Abdulrahman Bin Faisal University, Dammam City 31441, Saudi Arabia; 8 Department of Biology, Faculty of Science, Princess Nourah Bint Abdelrahman University, Riyadh 11451, Saudi Arabia; mfelkhadragy@pnu.edu.sa

**Keywords:** myiasis, prevalence, *Oestrus ovis*, *mtCOI*, sheep, goats, Saudi Arabia

## Abstract

**Simple Summary:**

In the current study, we investigated the prevalence rate of *Oestrus ovis* (*O. ovis*) larvae in heads of sheep and goats obtained from abattoirs in Riyadh, Saudi Arabia. It was found that season did not have any significant effect on the characteristics of the larvae that affect sheep, except for the length of larva, for which those collected in spring differed from those collected in winter and autumn. In goats, the L3 instar number was significantly higher during summer. *Oestrus ovis* larvae were detected in sheep and goats by molecular analysis and they were most closely related to *O. ovis* larvae recovered from GenBank.

**Abstract:**

Heads of sheep (*n* = 600) and goats (*n* = 800) slaughtered at Al-Aziziah Abattoir in Riyadh, Saudi Arabia, were inspected for the presence of *O. ovis* larvae (L). Heads were split along the longitudinal axes, and larvae (L1, L2, and L3) were gathered. The infestation rate was significantly higher in goats (44.5%; 356/800) than that in sheep (22.3%; 134/600). Out of the 151 collected larvae from sheep, 0% were L1, 1.3% were L2, and 98.7% were L3. Out of the total of 468 larvae from goats, 0% were L1, 1.2% were L2, and 98.8% were L3. The infestation rate was significantly higher in males than that in females. Myiasis-causing larvae collected from Riyadh, Saudi Arabia, were authenticated as *O. ovis*, according to morphological characteristics. Polymerase chain reaction (PCR) amplification of a partial fragment (600 bp) of the mitochondrial cytochrome c oxidase subunit I (*mtCOI*) gene further confirmed the species. Phylogenetic analysis based on the partial *mtCOI* gene sequence demonstrated that 23 unique sequences showed high similarity based on nucleotide pairs of *O. ovis* accessions retrieved from GenBank.

## 1. Introduction

The sheep gadfly *O. ovis* Linnaeus 1761 (Diptera, Oestridae) is one cause of myiasis in sheep and goats. Sneezing and nasal discharge are the most prominent clinical symptoms of infested animals [1]. Myiasis lowers the health of animals and causes major economic losses to the livestock industry due to abortion, reduced milk production, loss of weight gain, and fertility [2]. *Oestrus ovis* larvae persist in cranial cavities of sheep [3]. The incidence of *O. ovis*-related myiasis in the Jazan area was previously reported to be as high as 53.54% [1]. Clinical appearance, morphological characterizations of the larvae (e.g., slits of the posterior spiracles found on the posterior spiracle plates), and occasional identification of the adult fly are among the many approaches leading to the diagnosis of myiasis [4]. Molecular methods facilitate in the diagnosis and detection of a wide variety of species, including flies that cause myiasis [5,6]. Molecular approaches can be efficiently used to identify all larval stages [7]. Multiple genetic markers of ribosomal DNA (16S rRNA, 28S rRNA) and mitochondrial DNA (cytochrome oxidase genes (*COI*, *COII*, and 12S mtDNA)) have been used [8]. A widely used method for the molecular identification of myiasis-causing larvae belonging to the Oestridae family is the sequencing of the mitochondrial cytochrome c oxidase subunit I (*mtCOI*) gene, which contributes to an essential database for molecular identification of organisms [9,10]. Phylogenetic relationships can be assessed by polymerase chain reaction (PCR) and PCR restriction fragment length polymorphism (PCR-RFLP) assays [7]. For some species belonging to Calliphoridae, Sarcophagidae, and Oestridae, the mitochondrial gene subunit has been isolated, proving effective for molecular phylogenetic studies [4,11,12,13,14,15,16]. *Oestrus* spp. larvae identification can be complicated, as there are considerable morphological similarities within the species. The objectives of the present study were to investigate the seasonal prevalence of *O. ovis* among slaughtered sheep and goats in Riyadh, Saudi Arabia, and to use the sequencing of the *mtCOI* gene to identify the phylogenies of *O. ovis* larvae. One genetic marker, *COI*, was used to identify larvae collected from domestic sheep and goats to accurately identify *Oestrus* parasites [17]. 

## 2. Materials and Methods

### 2.1. Larvae Collection and Morphological Identification

This study was conducted between March 2019 and January 2020 in the city of Riyadh, Saudi Arabia. Riyadh City is in the center of the Kingdom of Saudi Arabia. It extends between latitude: 24°38′ North and longitude:46°43′ East. It has a very dry and arid climate; summer temperatures rise very dramatically during the day but fall at night, ranging between 35 °C and 43 °C. In the winter, the temperature drops dramatically and may reach 0 °C. Riyadh City is located 600 m above sea level, with several valleys and rich sand dunes. A total of 600 sheep and 800 goat heads from the Al-Aziziah Abattoir were analyzed for *O. ovis* larvae. The sex and age (<1 year is young and >1 year old is adult) of slaughtered animals were registered. At the investigation site, the nasal and frontal sinuses were longitudinally divided into the dorsoventral planes using a sharp handsaw. *Oestrus* *ovis* present in the mucous membrane of the nasal septa and nasal segments were collected with forceps and preserved in 70% ethanol for molecular studies and morphometric identification. Larvae were identified as the first, second, and third larval stages [18,19]. The percentage of each larval instar was calculated according to the equation (Larval stage percent = number of each larval stage/Total number of larvae × 100). In each infested head, the larvae were counted with the aid of identification keys [20]. The severity of the infestation was calculated as the number of larvae/number of animals infested. 

### 2.2. DNA Extraction and Polymerase Chain Reaction (PCR)

DNA from each individual larva (about 0.025 gm from anterior section of the larva) was extracted using a DNeasy Blood & Tissue Kit (250) (Cat No./ID: 69506, Qiagen, Hilden, Germany) following the manufacturers’ protocol. *mtCOI* gene was amplified using the primer pairs described in Table 1 [21] and a thermal cycler (Veriti^®^ 96-Well Thermal Cycler, Model 9902, Biosystem). This procedure was performed in a mixture (20 μL) consisting of master mix (5×, 4 μL), RNase-free water (12 μL), and DNA template (2 μL). The PCR program consisted of a denaturation step of 94 °C for 2 min, followed by 40 cycles of denaturation for 30 s at 94 °C. PCR products were analyzed by 1.5% agarose gel electrophoresis.

### 2.3. Nucleotide Sequences of the mtCOI Gene

A total of 40 sequences (Table S1), which were coded according to properties of their host, were analyzed in this study. The sequences were processed using Geneious Prime Build 2020-04-07 08:42 [22]. Prior to processing, all sequences were truncated using the error probability method with a limit of 0.05 on both sides. Related sequences were retrieved from GenBank after performing a BLAST [23] search. Multiple sequence alignments were then generated using CLUSTAL Omega [24] implemented in Geneious software. A phylogenetic tree was created using the neighbor-joining method [25] with the Tamura_Nei model and using 10,000 replicates. All samples were deposited to GenBank, and accession numbers have been provided.

### 2.4. Statistical Analysis

All data concerning the effect of different seasons were analyzed using one-way analysis of variance (ANOVA), followed by Duncan’s test to compare means. Statistical analysis was conducted using SPSS software program version 24, and all results were expressed as means ± SEM. Differences were considered significant at *p* ≤ 0.05.

## 3. Results

### 3.1. Prevalence of Infestation over the Course of the Study in Sheep and Goats

This study, which was conducted on 600 sheep and 800 goats (Table 2), showed that infestation rate with larvae was much higher in goats (44.5%; 356/800) than that in sheep (22.3%; 134/600). This also applied to the total number of larvae, where the total number of larvae in 356 head of goats was 486, while it was 151 in 134 head of sheep.

Results of statistical analysis concerning infestation rate showed that the ovine species is significantly more susceptible to infection with larvae than caprine species: the infestation mean in sheep was 1.78, and in goats was 1.56 at *p* ≤ 0.05, as a significant level.

**Figure 1:**
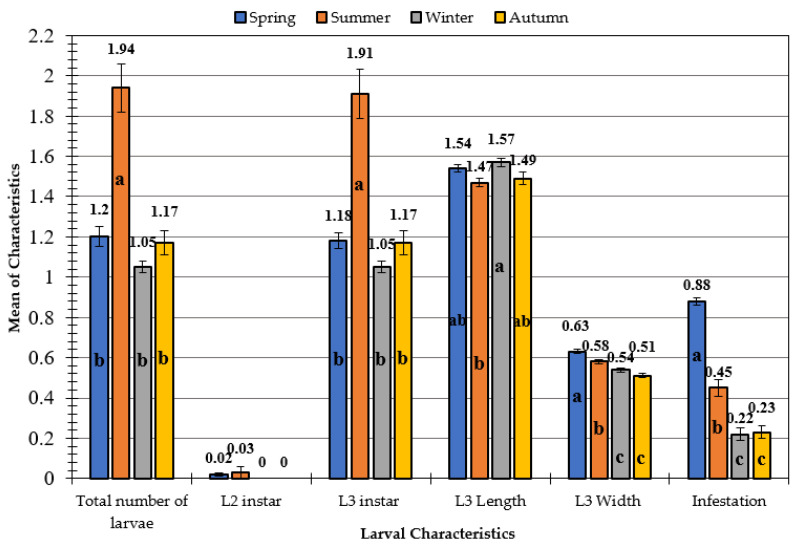
Effect of season on larval characteristics in infested goats. Mean values with different letters (a, b, c) are significantly different at *p* ≤ 0.05 between seasons.

The findings in Table 3 show that, in both species (goats and sheep), there was a disparity in the percentage of infestation between males and females, where the rate of infestation in males was higher than that in females (32.66% (98/300) and 65.50% (262/400) in sheep and goats, respectively). Additionally, the incidence of infestation was higher in female goats (32.50 percent; 94/400) than in female sheep (12.0 percent; 36/300). The seasonal effect on the characteristics of larvae specifically infesting goats (Figure 1) showed that there was a significant difference in the total number of larvae and L3 instar. The mean values of total larvae and L3 instar were significantly higher in the summer (1.94 ± 0.12 and 1.91 ± 0.12, respectively) than in other seasons.

**Figure 2:**
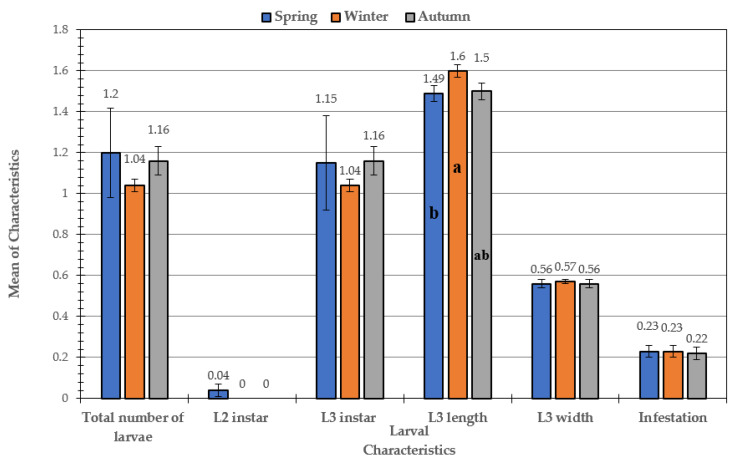
Effect of seasons on larval characteristics in infested sheep. Mean values with different letters (a and b) are significantly different at *p* ≤ 0.05 between seasons.

In addition, it was found that there was no significant difference between the means of total number of larvae and L3 in the spring, winter, and autumn. Concerning the larvae width and infestation, the trend was the same in the two characteristics. No significant difference between the winter and autumn was observed, while the significant differences in width and infestation appeared between spring and summer. As for the larvae length characteristic, it was found that only the winter and summer seasons varied significantly, while there were no significant variations between the spring and autumn seasons. The results of the impact of season on the studied characteristics differed only in goats. It was found that seasons of the year did not have any significant effect on the characteristics of the larvae that affect sheep, in general, except for the length of larva, in which those collected in spring differed from those collected in winter and autumn (Figure 2).

### 3.2. Morphological Examination

**Figure 3:**
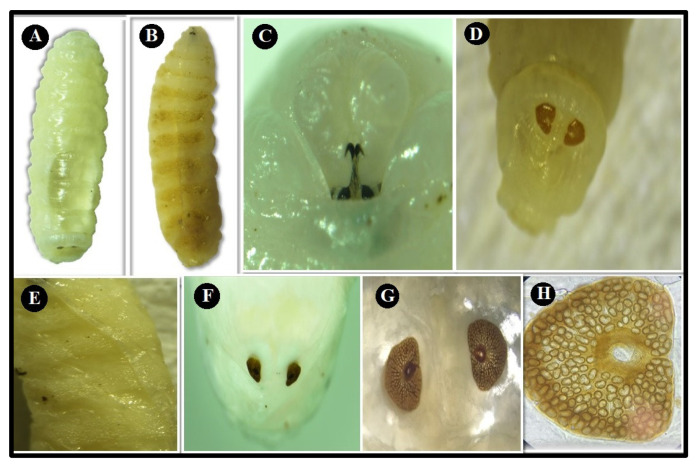
Second instar larva (L2). (**A**) Dorsal view; (**B**) ventral view; (**C**) the front end containing the cephaloskeleton ; (**D**) the median part of the postanal bulge is spinulose; (**E**) ventral view shows spines on anterior segment ; (**F**) posterior spiracle; (**G**) stigmal plates and channel appear with clear distinct suture; (**H**) posterior spiracle (10×).

The identification of mature *O. ovis* larvae was based on morphological characteristic features of the larval stages. Second instar larvae (L2) were found only in young male goats in spring and summer; the average length and width were 0.84 ± 0.08 cm and 0.26 ± 0.05 cm, respectively, in spring, while the average length and width were 0.90 ± 0.10 cm and 0.30 ± 0.00 cm, respectively, in the summer. These L2 larvae are white in color, and the dorsal side shows only a few weak denticles on the second segment (Figure 3A). On the ventral side, the segments have rows of peculiarly shaped currycomb-like spines (Figure 3B). Antennary lobes are less separated giving to pseudocephalon triangular shape. The buccal funnel is well structured. The hooks of the first segment are less robust and more curved (Figure 3C). Furthermore, the median part of the postanal bulge is spinulose (Figure 3D). Ventrally the segments are provided with rows of peculiarly shaped currycomb-like spines, the thoracic and abdominal segments on the ventral side, show single-ended, caudally projected spines (Figure 3E). The posterior peritremes are more or less circular (Figure 3F), with the channel indicated by a distinct suture (Figure 3G,H).

**Figure 4:**
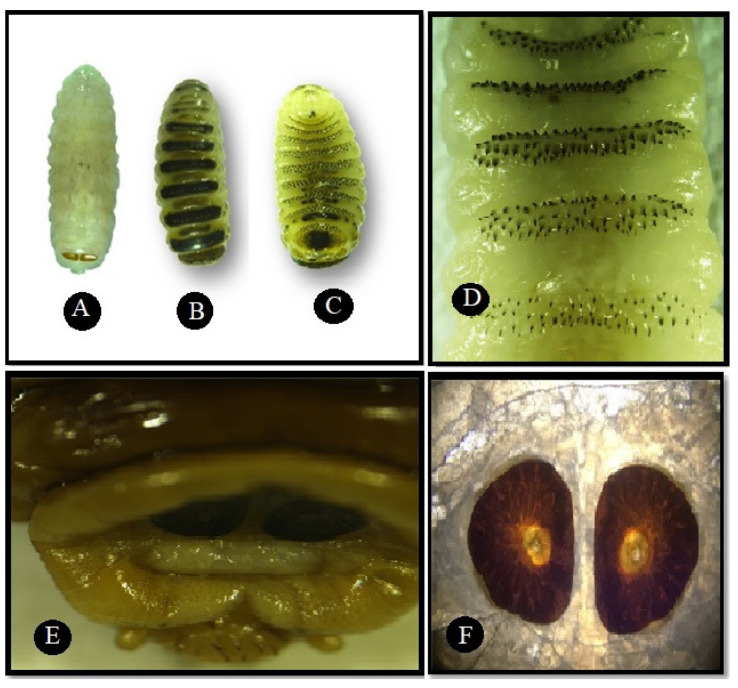
Third instar larva (L3). (**A**) Yellow color; (**B**) dorsal view shows broad transverse blackish bands; (**C**) ventral view shows spines on segments; (**D**) magnified view of the spines on the ventral segments; (**E**) postanal bulge shows fewer spines; (**F**) posterior spiracle is in a D-shape with central button but no distinct suture.

Third instar larvae (L3) are yellow in color when young (Figure 4A), changing to a light brown later in the mature stage, and show broad transverse blackish bands on the dorsal side (Figure 4B). The second segment on the dorsal side presents with a variable number of small denticles; the following segments are bare but have a rough, leatherlike skin pattern, which is distinct only on the darkened parts. On the ventral side, the segments bear rows of strong spines (Figure 4C), which are irregularly placed on the third segment but are fairly regular on the following ones. The number of rows varies from two to five (Figure 4D). The postanal bulge shows fewer spines, while the preanal bulge is bare (Figure 4E). The posterior peritremes are circular, with a central button and lacking a distinct suture and the channel appears in the posterior peritremes (Figure 4F).

### 3.3. PCR Amplification and Nucleotide Sequence Analysis of the Partial mtCOI Gene

**Figure 5:**
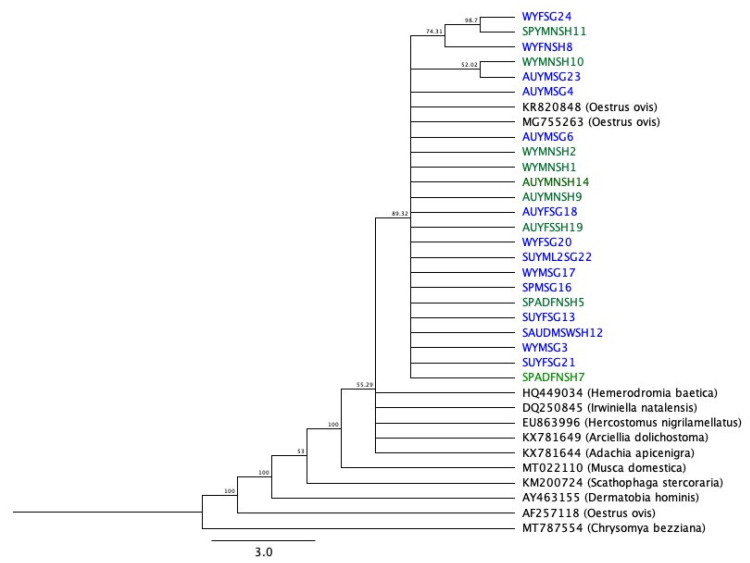
Genetic relationships of the *mtCOI* gene collected samples with other species retrieved from GenBank. The tree is generated using the neighbor-joining method, implemented in the Geneious software, with the Tamura_Nei model.

PCR amplification was performed using a set of primers (FFCOI and RHCO) to amplify a 600-bp fragment of the *mtCOI* gene from *O.* *ovis* larvae. A total of 23/40 sequences were analyzed in this study (sequences with HQ% (percent high quality) score less than 40 were discarded). The pairwise identity of sheep sequences was 97.9%, and that of goat sequences was 99.2%. Overall, the 23 sequences shared 98.1% pairwise identity and had 35.6% GC content. The results of BLAST search showed that all the sequences had high similarity to the *O. ovis* “sheep botfly” in GenBank. Figure 5 shows the phylogenetic tree indicating respective evolutionary relationships. MT787554 (*Chrysomya bezziana*) is used as an out-group. 

## 4. Discussion

Out of the examined 600 sheep and 800 goat heads, 22.3% and 44.5%, respectively, were infested with *O. ovis* larvae. These percentages were high compared with previous studies in Saudi Arabia [26], which recorded 5.9% in Riyadh Region and 0.299% from adult *O. ovis* spp. in Jeddah [27], but similar to the 53.5% (257/480) reported in the Jazan Region [1]. Our results were also similar to those reported in Greece (43.2%) [28], Turkey (22.52%) [29], and Libya (42.33%) [30]. Our results were higher than those reported by other researchers around the world, such as by [31] in Egypt (8.67%), but less than those in Spain (71.1%) [32] and Italy (91.0%) [33]. These differences may be a result of differences in geographical area, animal breed, and climate. The infestation rate was higher in male sheep and goats than females (16.35% and 32.75%, respectively, compared to 6% and 11.75%; *p ≤* 0.05). This was consistent with a report investigating sex differences in infestation in Libya [30]. 

In the central region of Saudi Arabia, the spring months are the best time of the year, with low temperature and humidity [26]. The disappearance of fly activity in most of the year is probably due to the hot, dry, and windy weather. The 27 °C was optimum temperature for adults emerge from pupa, while constant temperatures below 16 °C and above 32 °C were fatal [34].

Our results did not demonstrate any significant seasonal prevalence of infestation in sheep, suggesting that time of the year does not have any significant effect. This is in disagreement with a previous study [35], which mentioned that the peaks of infestation in Turkey were in the summer and spring, while the lowest infestation rates were in autumn. Another study in Egypt [36] concluded that heavier infestations were recorded in autumn (17.91%), while the lowest seasonal peak was in winter (7.85%). In Iraq, the highest recorded range was in the autumn, which was 76.92%, and the lowest range was in winter (30.76%) [37]. The reason for these differences may be changes in environmental conditions.

The present study recorded that 0% were L1, 1.5% were L2, and 111.2% were L3 of the total of 151 larvae collected from sheep, and 0% were L1, 1.7% were L2, and 135.4% were L3 of the of total 468 larvae collected from goats. The L1 result was lower than that recorded in previous studies [30,35] that reported that L1 larvae prevailed at 90.5% of the overall weight, and another study [37] that reported 13.14% L1 larvae. Our results were in agreement with one study [38] that reported a higher level of larvae in stage L3 (63.4%) than that in L2 (26.6%) and L1 (10%). It is possible that L1 larvae might have passed unseen due to their small size, that they were present in hidden places such as turbinates and ethmoid bones, or that numerous L1 were demolished in the nasal holes during the hypo-biotic period [39]. The morphological characters of L2 and L3 were in accordance with those stated in the identification key [20].

PCR analysis was performed using set of primers (FFCOI and RHCO) and successfully amplified 606bp fragment of *mtCOI* gene from *O.ovis* larvae. The sequence of amplified fragment showed genome arrangement typical of *O. ovis* sequence in Genbank. PCR amplification of *mtCOI* gene from eighteen species of Oestridae causing myiasis resulted in 686 bp [12]. Rooted phylogenetic trees elucidated phylogenetic relationships between *O.ovis* larvae amplicons and other members of Family: Oestridae published in Genbank.

The results of BLAST search showed that all the sequences had high similarity to the *O. ovis* “sheep botfly” identified in Brazil (accession number, KR820703) [40], with pairwise identity between 94.8% and 99.5% and coverage more than 86%. The sequences are also similar to the *O*. *ovis* sheep botfly identified in Turkey (accession number, MT124626) [41], with pairwise identity between 94.2% and 98.70% and coverage greater than 86.18%.

## 5. Conclusions

In this study, the results indicated that the infestation rate was significantly higher in sheep than goat, male than female, and spring season than other seasons of the year.

## Figures and Tables

**Table 1:** Set of primers used for the amplification of mitochondrial cytochrome c oxidase subunit I (*mtCOI*) gene using extracted DNA of *O. ovis* larvae.

Gene	Primers	Sequences	References
*mtCOI*	FFCOI	5′-GGAGCATTAATYGGRGAYG-3′	[21]
	RHCO	5′-TAAACTTCAGGGTGACCAAAAATCA-3′	

**Table 2:** Prevalence of *Oestrus* larvae in sheep and goats over the course of the study.

Species	No. of Head Examined	Infestation Rate (%)	Mean of Infestation	No. of Larvae (%)			Total Larvae (%)
				L1	L2	L3	
Sheep	600	134 (22.3)	1.78 ± 0.02 ^a^	0.0 (0.0)	2.0 (1.5)	149.0 (111.2)	151.0 (112.7)
Goat	800	356 (44.5)	1.56 ± 0.02 ^b^	0.0 (0.0)	6.0 (1.7)	482.0 (135.4)	486 (136.5)

Mean values with different superscript in column differed significantly at *p* ≤ 0.05.

**Table 3:** Sex differences on the prevalence of *Oestrus* larvae in sheep and goats.

Species	Sex	No. of Examined Animals	No. of Infested (%)
Sheep	Male	300	98 (32.66)
	Female	300	36 (12)
Goat	Male	400	262 (65.5)
	Female	400	94 (32.5)
Total		1400	490 (35.00)

## Data Availability

All relevant data are within the paper.

## Supplementary Materials

The following are available online at https://www.mdpi.com/2076-2615/11/3/689/s1, Table S1: Accession numbers of samples deposited to GenBank.

## References

[1] Hanan B.A. (2013). Seasonal prevalence of *Oestrus ovis* L. (Diptera: Oestridae) larvae in infested sheep in Jazan Region. Saudi Arabia. J. Parasitol. Vector Biol..

[2] Traversa D., Otranto D. (2006). A new approach for the diagnosis of myiasis of animals: The example of horse nasal myiasis. Vet. Parasitol..

[3] Hidalgo A., Palma H., Oberg C., Fonseca-Salamanca F. (2015). *Oestrus ovis* infestation of grazing sheep during summer in southern Chile. Pesqui. Veterinária Bras..

[4] Ogo N.I., Onovoh E., Okubanjo O.O., Galindo R.C., De La Lastra J.-M.P., De La Fuente J., De La Lastra J.M.P. (2012). Molecular identification of *Cordylobia anthropophaga Blanchard* (Diptera: Calliphoridae) larvae collected from dogs (*Canis familiaris*) in Jos South, Plateau State, Nigeria. Onderstepoort J. Vet. Res..

[5] Otranto D., Traversa D. (2004). Molecular evidence indicating that *Przhevalskiana silenus*, *P. aegagri* and *P. crossii* (Diptera, Oestridae) are one species. Acta Parasitol..

[6] Hall M., Adams Z., Wyatt N., Testa J., Edge W., Nikolausz M., Farkas R., Ready P. (2009). Morphological and mitochondrial DNA characters for identification and phylogenetic analysis of the myiasis causing flesh fly *Wohlfahrtia magnifica* and its relatives, with a description of *Wohlfahrtia monegrosensis* sp. n. Wyatt & Hall. Med. Vet. Entomol..

[7] Ames C., Turner B., Daniel B. (2006). The use of mitochondrial cytochrome oxidase I gene (COI) to differentiate two UK blowfly species—*Calliphora vicina* and *Calliphora vomitoria*. Forensic Sci. Int..

[8] Otranto D., Stevens J.R. (2002). Molecular approaches to the study of myiasis-causing larvae. Int. J. Parasitol..

[9] Otranto D., Puccini V. (2000). Cytochrome oxidase I (COI) gene of some obligate myiasis causing larvae (Diptera: Oestridae): Which perspective for phylogenetic studies? Preliminary data. Proc. Cost Action.

[10] Otranto D., Colwell D.D., Milillo P., Di Marco V., Paradies P., Napoli C., Giannetto S. (2004). Report in Europe of nasal myiasis by *Rhinoestrus* spp. in horses and donkeys: Seasonal patterns and taxonomical considerations. Vet. Parasitol..

[11] Otranto D., Colwell D.D., Traversa D., Stevens J.R. (2003). Species identification of *Hypoderma* affecting domestic and wild ruminants by morphological and molecular characterization. Med. Vet. Entomol..

[12] Otranto D., Traversa D., Guida B., Tarsitano E., Fiorente P., Stevens J.R. (2003). Molecular characterization of the mitochondrial cytochrome oxidase I gene of Oestridae species causing obligate myiasis. Med. Vet. Entomol..

[13] Otranto D., Milillo P., Traversa D., Colwell D.D. (2005). Morphological variability and genetic identity in *Rhinoestrus* spp. causing horse nasal myiasis. Med. Vet. Entomol..

[14] Li X., Cai J.F., Guo Y.D., Wu K.L., Wang J.F., Liu Q.L., Wang X.H., Chang Y.F., Yang L., Lan L.M. (2010). The availability of 16S rRNA for the identification of forensically important flies (Diptera: Muscidae) in China. Trop. Biomed..

[15] Xinghua W., Jufeng C., Yadong G., Yufeng C., Kunlu W., Qinlai L., Jifeng C., Yunfeng C., Jiangfeng W., Yang L. (2010). The availability of 16SrDNA gene for identifying forensically important blowflies in China. Rom. J. Leg. Med..

[16] Hendawy S.H., Allam N.A., Kandil O.M., Zayed A.A., Desouky AR A., Mahmoud A. (2012). Partial COI and 16S rRNA genes sequences of *Cephalopina titillator* mitochondrial dna: Evidence for variation in evolutionary rates within myiasis causing species. Indian J. Anim. Res..

[17] Moreno V., Romero-Fernández I., Marchal J.A., Beltran M., Granados J.E., Habela M.A., Tamadon A., Rakhshandehroo E., Sarasa M., Pérez J.M. (2015). Molecular characterization of bot flies, *Oestrus* spp., (Diptera, Oestridae), from domestic and wild Bovidae hosts. Vet. Parasitol..

[18] Ferrar P. (1987). A Guide to the Breeding Habits and Immature Stages of Diptera Cyclorrhapha.

[19] Smith K.G. (1989). An Introduction to the Immature Stages of British Flies: Diptera Larvae, with Notes on Eggs, Puparia and Pupae.

[20] Zumpt F. (1965). Myiasis in man and animals in the Old World. A textbook for physicians, veterinarians and zoologists. Myiasis in Man and Animals in the Old World: A Textbook for Physicians, Veterinarians and Zoologists.

[21] Bosly A.H. (2018). Molecular identification studies on *Oestrus ovis* L. (Diptera: Oestridae) larvae infested sheep in jazan region, Saudi Arabia. Indian J. Anim. Res..

[22] Drummond A.J., Ashton B., Buxton S., Cheung M., Cooper A., Duran C., Field M., Heled J., Kearse M., Markowitz S. (2010). Geneious v5.3. Biomatters Ltd: Auckland, New Zealand. http://www.geneious.com.

[23] Altschul S.F., Gish W., Miller W., Myers E.W., Lipman D.J. (1990). Basic local alignment search tool. J. Mol. Biol..

[24] Sievers F., Wilm A., Dineen D., Gibson T.J., Karplus K., Li W., Lopez R., McWilliam H., Remmert M., Söding J. (2011). Fast, scalable generation of high-quality protein multiple sequence alignments using Clustal Omega. Mol. Syst. Biol..

[25] Saitou N., Nei M. (1987). The neighbor-joining method: A new method for reconstructing phylogenetic trees. Mol. Biol. Evol..

[26] Alahmed A.M. (2000). Seasonal infestation of *Oestrus ovis* larvae in sheep heads in central region of Saudi Arabia. J. Egypt. Soc. Parasitol..

[27] Alikhan M., Al Ghamdi K., Al Zahrani F.S., Khater E.I., Allam A.M. (2018). Prevalence and Salient Morphological Features of Myiasis Causing Dipteran Flies in Jeddah, Saudi Arabia. Biosci. Biotechnol. Res. Asia.

[28] Papadopoulos E., Chaligiannis I., Morgan E.R. (2010). Epidemiology of *Oestrus ovis* L. (Diptera: Oestridae) larvae in sheep and goats in Greece. Small Rumin. Res..

[29] Karatepe B., Karatepe M., Güler S. (2014). Epidemiology of *Oestrus ovis* L. infestation in sheep in Nigde province, Turkey. Rev. Médecine Vétérinaire.

[30] Mohsen M.N., Raham S.E., Gasim M.H. (2015). *Oestrus ovis* larval infestation among sheep and goats of green moun-tain areas in Libya. J. Adv. Vet. Anim. Res..

[31] Amin A.R., Morsy T.A., Shoukry A., Mazyad S.A. (1997). Oestrid head maggots in slaughtered sheep in Cairo abattoir. J. Egypt. Soc. Parasitol..

[32] Alcaide M., Reina D., Sánchez J., Frontera E., Navarrete I. (2003). Seasonal variations in the larval burden distribution of *Oestrus ovis* in sheep in the southwest of Spain. Vet. Parasitol..

[33] Caracappa S., Rilli S., Zanghi P., Di Marco V., Dorchies P. (2000). Epidemiology of ovine oestrosis (*Oestrus ovis* Linné 1761, Diptera: Oestridæ) in Sicily. Vet. Parasitol..

[34] Rogers C.E., Knapp F.W. (1973). Bionomics of the Sheep Bot Fly, *Oestrus Ovis*. Environ. Entomol..

[35] Arslan M.O., Kara M., Gicik Y. (2009). Epidemiology of Oestrus ovis infestations in sheep in Kars province of north-eastern Turkey. Trop. Anim. Health Prod..

[36] Ramadan M.Y., Khater H.F., Omer S.F., Rahman A.A. Epidemiology of *Oestrus ovis* infesting Egyptian sheep. Proceedings of the XX International Congress of Mediterranean Federation of Health and Production of Ruminants.

[37] Mohammed R.G., Josef S.S., Abed K.J. (2020). Prevalence of *Oestrus ovis* Larvae in Slaughtered Sheep of Misan City, Iraq. Syst. Rev. Pharm..

[38] Yilma J., Dorchies P. (1991). Epidemiology of *Oestrus ovis* in southwest France. Vet. Parasitol..

[39] Bart A.G., Minár J. (1992). Probability description of regulation on the level of population and individual in the host-parasite system using *Oestrus ovis* (Diptera: Oestridae) as an example. Folia Parasitol..

[40] Marinho M.A.T., Wolff M., Ramos-Pastrana Y., de Azeredo-Espin A.M.L., Amorim D.D.S. (2017). The first phylo-genetic study of Mesembrinellidae (Diptera: Oestroidea) based on molecular data: Clades and congruence with morpholog-ical characters. Cladistics.

[41] Karademir G.K., Usluğ S., Okur M., Inci A., Yıldırım A. (2020). Molecular Characterization and Phylogenetic Analyses of *Oestrus ovis* Larvae Causing Human Naso-pharyngeal Myiasis Based on CO1 Barcode Sequences. Turk. J. Parasitol..

