# Supplementary material for: Prevalence Rate and Molecular Characteristics of Oestrus ovis L. (Diptera, Oestridae) in Sheep and Goats from Riyadh, Saudi Arabia
Source: Animals (Basel). 2021 Mar 4;11(3):689. doi: 10.3390/ani11030689 (PMC7999880; doi:10.3390/ani11030689)
Supplement: Supplementary file 1 [file animals-11-00689-s001.pdf]

animals

MDPI

Table S1. Accession numbers of samples deposited to GenBank.

No.	Accession Numbers	Description
1	SpYMNSh	Spring, Young , Male, Niemi, Sheep
2	SpAdFNSh	Spring, Adult, Female, Niemi, Sheep
3	SpyFL2NSh	Spring, Young, Female, L2 stage larva, Niemi, Sheep
4	SpAdFNSh	Spring, Adult, Female, Niemi, Sheep
5	SpAdMNSh	Spring, Adult, Male, Niemi, Sheep
6	SpYMSG	Spring, Young, Male, Somali, Goat
7	SpAdMSG	Spring, Adult, Male, Somali, Goat
8	SpyML2SG	Spring, Young, Male, L2 stage larva, Somali, Goat
9	SpAdFSG	Spring, Adult, Female, Somali, Goat
10	SpMSG	Spring, Young, Male, Somali, Goat
11	SuAdMSwSh	Summer, Adult, Male, Swakni, Sheep
12	SuAdMSwSh	Summer, Adult, Male, Swakni, Sheep
13	SuyFSG	Summer, Young, Female, Somali, Goat
14	SuyMSG	Summer, Young, Male, Somali, Goat
15	SuyML2SG	Summer, Young, Male, L2 stage larva, Somalia, Goat
16	SuyFSG	Summer, Young, Female, Somali, goat
17	SuyFSG	Summer, Young, Female, Somali, Goat
18	SuyMSG	Summer, Young, Male, Somali, Goat
19	SuyMSG	Summer, Young, Male, Somali, Goat
20	AuyMNSh	Autumn, Young, Male, Niemi, Goat
21	AuyFSSh	Autumn, Young, Female, Somali, Sheep
22	AuyFNSh	Autumn, Young, Female, Niemi, Sheep
23	AuyFSSh	Autumn, Young, Female, Somali, Sheep
24	AuyMNSh	Autumn, Young, Male, Niemi, Sheep
25	AuyMSSh	Autumn, Young, Male, Somali, Sheep
26	AuyMSG	Autumn, Young, Male, Somali, Sheep
27	AuyFSG	Autumn, Young, Female, Somali, Goat
28	AuyMSG	Autumn, Young, Male, Somali, Goat
29	AuyMSG	Autumn, Young, Male, Somali, Goat
31	WyMNSh	Winter, Young, Male, Niemi, Sheep
32	WyFNSh	Winter, Young, Female, Niemi, Sheep
33	WyFNSh	Winter, Young, Female, Niemi, Sheep
34	WyMNSh	Winter, Young, Male, Niemi, Sheep
35	WyMNSh	Winter, Young, Male, Niemi, Sheep
36	WyMSG	Winter, Young, Male, Somali, Goat
37	WyFSG	Winter, Young, Female, Somali, Goat
38	WyFSG	Winter, Young, Female, Somali, Goat
39	WyMSG	Winter, Young, Male, Somali, Goat
40	WyMSG	Winter, Young, Male, Somali, Goat
